# Co-infection with *Fasciola hepatica* may increase the risk of *Escherichia coli* O157 shedding in British cattle destined for the food chain

**DOI:** 10.1016/j.prevetmed.2017.12.007

**Published:** 2018-02-01

**Authors:** Alison K. Howell, Sue C. Tongue, Carol Currie, Judith Evans, Diana J.L. Williams, Tom N. McNeilly

**Affiliations:** a Department of Infection Biology, Institute of Infection and Global Health, University of Liverpool, Liverpool, L69 7ZJ, UK; b SRUC Research, Epidemiology Research Unit, (Inverness Campus), Scotland’s Rural College (SRUC), Kings Buildings, West Mains Road, Edinburgh, EH9 3JG, UK; c Moredun Research Institute, Pentlands Science Park, Bush Loan, Penicuik, Midlothian, EH26 0PZ, UK; d School of Veterinary Science, University of Liverpool, Leahurst, Chester High Road, Neston, CH64 7TE, UK

**Keywords:** Escherichia coli O157, Fasciola hepatica, Cattle, Co-infection

## Abstract

*Escherichia coli* O157 is a zoonotic bacterium that can cause haemorrhagic diarrhoea in humans and is of worldwide public health concern. Cattle are considered to be the main reservoir for human infection. *Fasciola hepatica* is a globally important parasite of ruminant livestock that is known to modulate its host’s immune response and affect susceptibility to bacterial pathogens such as *Salmonella* Dublin. Shedding of *E. coli* O157 is triggered by unknown events, but the immune system is thought to play a part. We investigated the hypothesis that shedding of *E. coli* O157 is associated with *F. hepatica* infection in cattle. Three hundred and thirty four cattle destined for the food chain, from 14 British farms, were tested between January and October 2015. *E. coli* O157 was detected by immunomagnetic separation and bacterial load enumerated. *F. hepatica* infection status was assessed by copro-antigen ELISA. A significant association (*p* *=* 0.01) was found between the log percent positivity (PP) of the *F. hepatica* copro-antigen ELISA and *E. coli* O157 shedding when the fixed effects of day of sampling and the age of the youngest animal in the group, plus the random effect of farm were adjusted for. The results should be interpreted cautiously due to the lower than predicted level of fluke infection in the animals sampled. Nevertheless these results indicate that control of *F. hepatica* infection may have an impact on the shedding of *E. coli* O157 in cattle destined for the human food chain.

## 1 Introduction

*Fasciola hepatica,* or the common liver fluke, is a parasite of ruminant livestock, occurring worldwide. Various studies have shown that *F. hepatica* can affect host immunity to other pathogens (Moreau and Chauvin, 2010), by making the host more susceptible to infection (Aitken et al., 1979, Aitken et al., 1981, Brady et al., 1999); changing the pathogenesis of disease (Garza-Cuartero et al., 2016); and interfering with diagnostic tests (DEFRA, 2005, Flynn et al., 2007). This happens because infection with *F. hepatica* induces a mixed T helper type-2 (Th2) and T-regulatory response, with increased production of IL4, IL5, IL10, IL13 and TGFβ, whilst T helper type-1 (Th1) responses are down regulated (Flynn et al., 2010, Graham-Brown, 2016).

*Escherichia coli* O157 is a zoonotic bacterium that occurs worldwide and can cause haemorrhagic diarrhoea in humans as a result of systemic Shiga toxin (Stx) activity. Cattle are considered the main source of human infection, either through direct contact or through contaminated food (Locking et al., 2001, Strachan et al., 2006, Gyles, 2007). An estimated 20–40% of British cattle herds are reported to shed *E. coli* O157 (Paiba et al., 2003, Ellis-Iversen et al., 2007, Gunn et al., 2007, Pearce et al., 2009). The annual reported incidence of human *E. coli* O157 is 1.8 culture positive cases per 100,000 population in England and Wales, and 4.5 cases per 100,000 in Scotland (Health Protection Network, 2013, Public Health England, 2013). In a proportion of cases, mainly in young children, haemolytic uraemic syndrome may occur, and is potentially fatal (Chase-Topping et al., 2008).

*Escherichia coli* O157 infections in cattle are usually asymptomatic as cattle lack vascular receptors for Stx (Pruimboom-Brees et al., 2000), but both cellular and humoral immune responses are induced and are required for immunity to *E. coli* O157 (Corbishley et al., 2014, Corbishley et al., 2016). Furthermore, cellular responses to *E. coli* O157 are associated with Th1 responses (Corbishley et al., 2014). The relationship between the shedding of *E. coli* and immunity is not fully understood, but shedding has been associated with stressful events that could affect the immune response (Ellis-Iversen et al., 2007, Munns et al., 2015).

Recent estimates using bulk milk antibody detection ELISAs based on fluke excretory-secretory antigens show 50–80% of UK dairy herds have been exposed to fluke (McCann et al., 2010, Howell et al., 2015). Although the current status for the beef sector is unknown, figures released by the Food Standards Agency report that 16.5% of cattle livers were condemned due to liver fluke during 2015 (Ford and Hadley, 2015). Since liver fluke infection down-regulates Th1 responses, which are associated with clearance of the bacteria from the bovine gut (Corbishley et al., 2014), we hypothesized that fluke infection could affect the propensity of cattle to shed *E. coli* O157. If so, the presence of co-infected cattle could increase the risk of zoonotic *E. coli* infections.

## 2 Methods

This study was designed to utilise samples collected for an existing larger study on *E. coli* O157 in cattle intended for human consumption, funded by Food Standards Scotland (FSS) and the Food Standards Agency (FSA; Project FS101055); referred to below as the FSS/FSA study. For the FSS/FSA study, sample size calculations showed that a minimum of 110 Scottish farms and 160 farms from England and Wales were required to estimate a prevalence of *E. coli* O157 of 20% and 35% respectively within a tolerance of 0.168 with 95% confidence (Henry et al., 2017).

A sample size calculation to determine the number of cattle that were required to investigate the association between *F. hepatica* infection and *E. coli* O157 shedding was performed by Hickey et al. (2015) using simulated datasets. The estimated prevalence of *E. coli* O157 was set at 4% of cattle and 20% of farms (Pearce et al., 2009) whilst the estimated prevalence of *F. hepatica* was set at 20% of cattle and 80% of farms (Salimi-Bejestani et al., 2005b, McCann et al., 2010). 100% sensitivity and specificity of both tests were assumed. The result of using these parameters was that the inclusion of 1645 individual samples, from 50 randomly selected farms, would give the study a power of 87% to detect a two-fold increase in the odds that an animal would shed *E. coli* O157 if it was also infected with *F. hepatica,* compared to cattle not infected with fluke.

### 2.1 Sample and data collection

Two hundred and seventy farms were sampled in the FSS/FSA study (Henry et al., 2017). These included a variety of types of enterprise and breeds of cattle. Of these, 110 were Scottish farms, randomly selected from all Scottish farms that had participated in both of two earlier studies. The inclusion criterion was that there was at least one male aged one year or over, or female over two without calves on the farm, as these farms were most likely to contain animals that would end up in the food chain. In addition, 160 farms for England and Wales were recruited from a randomly selected subset with either a male of any breed aged over 1 year, or a female of a non-dairy breed aged over 1 year. Farmers were initially notified by letter and given the choice to opt out, and were then contacted by phone in a randomised order to enrol them in the study.

Farms were visited once between September 2014 and November 2015. Individual fresh faecal pat samples were taken from the floor or ground, for the group of cattle from each farm that contained the animals closest to going off the farm for slaughter. The number of samples collected from each group was determined by a protocol assuming that if 8% of animals were positive, there would be a 0.9 probability of identifying groups containing at least one positive animal (Chase-Topping et al., 2007). It was assumed that a pat sample is equivalent to an animal level unit for analysis. These samples were then sent to the Epidemiology Research Unit (ERU) microbiological facilities at Scotland’s Rural College (SRUC), Inverness, within 48 h of collection, and tested for *E. coli* O157. The recruitment and visits were done by members of SRUC project team in Scotland, and the ADAS project team in England and Wales.

Farms for which samples were submitted to SRUC’s ERU laboratory on or after 5th January 2015, and which consented to further use of their samples and data for research purposes, were included in the study. Delays due to funding and contractual issues meant that samples received prior to this date were not retained.

Information on animal characteristics and farm management was collected from the livestock keeper or farm manager on each farm, via a questionnaire administered by the survey staff. The information was collected in an electronic format and was a shortened version of a questionnaire used in a previous study (Chase-Topping et al., 2007). The questionnaire was piloted with several farmers before use. The finalised questionnaire was approved by the FSS Survey Control team (Henry et al., 2017). The questionnaire was conducted in Welsh for Welsh-speaking respondents.

The information obtained was at the farm level, for example the age of animals was given as a range for the group, and all animals in a group were treated as having been managed the same in terms of housing, feeding and treatments given. The information relevant for the current study was identified and extracted. As the aim was to develop a model to determine the presence of an association between fluke and *E. coli* O157, rather than a predictive model, only management information relevant to fluke was taken for use in the model, to control for possible confounders which may be linked to both fluke and *E. coli* O157. A summary of these is shown in Table 1.

**Table 1:** Characteristics of the animals and farms in the fluke and *E. coli* O157 study.

		**Farm-level (n = 14)**	**Individual animals (n = 334)**
Date	Day of sample collection (Day 1 = 1 st Jan 2014)	Range = 20–293	
		Median = 126	
Housing at time of collection	Grazing	3 (21.43 %)	
	Housed	11 (78.57 %)	
Enterprise type	Dairy	2 (14.29 %)	
	Suckler beef	8 (57.14 %)	
	Finisher	2 (14.29 %)	
	Other	2 (14.29 %)	
Age	Youngest in group (months)	Range = 6–26	
		Median = 14.5	
	Oldest in group	Range = 11–48	
		Median = 20	
Herd size	Total number of cattle on farm	Range = 41–516	
		Median = 117	
Herd size	Total number of cows on farm	Range = 0–208	
	(Females that have had a calf)	Median = 33	
Herd size	Total number of heifers on farm	Range = 0–65	
		Median = 6	
Herd size	Total number of cattle under 1 year on farm	Range = 0–215	
		Median = 30	
Sheep	Total number of ewes on farm	Range = 0–700	
		Median = 0	
Sheep	Total number of sheep overwintering on farm	Range = 0–433	
		Median = 0	
Water supply	Water supply from mains	10 (71.43 %)	
	Water supply from spring or well	6 (42.86 %)	
	Water supply from natural source	11 (78.57 %)	
Fluke status	Median percentage of fluke positive cows^a^	6.55%	
	Range of positive cows	2.13–100%	
	Fluke positive		44 (13.17%)
	Median PP		0.82
	Range PP		−1.07–73.74
*E. coli* O157 status	Median percentage of E. coli O157 positive cows^b^	43.10%	
	Range of positive cows	4.00–100%	
	*E. coli* positive		170 (50.9%)
	Median cfu/g^c^		10
	Range cfu/g^c^		0−1.45 x 10^5^

a ‘Fluke positive’ refers to an animal which tested positive on the copro-antigen ELISA result.

b ‘*E*. *coli* O157 positive’ refers to an animal with a positive IMS *E*. *coli* test.

c Samples from which *E. coli* numbers fell below the limit of enumeration were assigned cfu/g = 10.

### 2.2 *E. coli* testing

One gram of faeces was added to 20 ml of buffered peptone water (BPW, Thermo Scientific, UK). The BPW was incubated for six hours at 37 °C (±1°) then subjected to immunomagnetic separation (IMS). Briefly, a 1 ml aliquot from each 20 ml BPW sample was added to 20 μl paramagnetic beads coated with polyclonal antibody for *E. coli* O157 lipopolysaccharide (Lab M Ltd., UK). The aliquots were mixed on a rotary mixer for 30 min before being washed three times in PBS with 0.05% Tween 20 (PBST, Sigma-Aldrich Co. Ltd.). After the third wash, the beads were re-suspended in 100 μl PBST and cultured onto MacConkey agar containing sorbitol, cefixime (0.05 mg/l) and tellurite (2.5 mg/l) (CT-SMac, Thermo Scientific, UK)(Jenkins et al., 2003).

Following overnight incubation at 37 °C (±1°) plates were examined for non-sorbitol-fermenting colonies and any suspect colonies were subcultured onto Chromocult coliform agar (Merck KGaA., Germany). After a further overnight incubation at 37 °C (±1°) any resulting red colonies were tested with anti-*E. coli* O157 latex (Thermo Scientific, UK) for agglutination. Colonies that agglutinated were identified as presumptively positive and enumerated by limiting dilution.

Polymerase Chain Reaction (PCR) was used to confirm the serogroup of the isolates as *E. coli* O157 (ISO/TS, 2012). For all positive samples, the number of *E. coli* O157 were enumerated by culturing 10-fold dilutions of faeces in minimum recovery diluent, starting from 1:10, on duplicate CT-SMac plates. Typical colonies were counted after overnight incubation at 37 °C (±1°) and counts expressed as colony forming units (cfu) per gram of faeces.

IMS is considered to be a highly sensitive and specific method of identifying *E. coli* O157, and has a lower limit of detection of 50 cfu/g (Aydin et al., 2014, Wright et al., 1994). Lower cfu counts can be detected with decreased sensitivity. IMS has a specificity of 99% (Ekong et al., 2017), and all positive isolates were confirmed as such by the Scottish *E. coli* Reference Laboratory. For the positive/negative analysis, an *E. coli* O157 positive cow was defined as one that tested positive by IMS. The limit of accurate enumeration was 100 cfu/g of faeces (Pearce et al., 2004), and samples from which too few *E. coli* were cultured to be enumerated were assigned a cfu/g of 10.

### 2.3 *F. hepatica* testing

Extraneous faecal material (2 g), from each faecal sample was weighed into polypropylene tubes and frozen (–20 °C). When the *E. coli* O157 status of the farms was known (as defined in Henry et al., 2017), all the samples from eligible *E. coli* O157 positive farms were transported to Moredun Research Institute (MRI) in batches. Here they were tested using a copro-antigen ELISA according to the manufacturer’s instructions (Bio-X Diagnostics, Jemelle, Belgium). MRI staff members were blinded to the *E. coli* O157 status of the individual samples. Freezing the samples prior to performing the copro-antigen ELISA is reported to make no difference to the sensitivity or specificity of the test (Brockwell et al., 2013, Flanagan et al., 2011), and this was also confirmed before this study commenced (Personal communication, Dr Philip Skuce).

The result was determined by calculating the percentage positivity (PP) of each sample relative to the optical density (OD) of the positive control, after subtracting the OD of the negative control (provided in the kit). The positive/negative cut off was determined by the quality control insert supplied with the kit, and was either 7 or 8 for all the kits used for this study. This test has a reported sensitivity of 0.77 and a specificity of 0.99 (Mazeri et al., 2016).

### 2.4 Statistical analysis

The epidemiological unit of interest was the individual animal. For each animal for which a sample was tested, the following results were obtained: *E. coli* O157 positive/negative, *E. coli* O157 cfu/g, *F. hepatica* positive/negative derived by applying the cut off to the copro-antigen ELISA results, and *F. hepatica* PP result (on a continuous scale). Farms without a single fluke positive animal were excluded from further analysis, to ensure that cattle at least had a possibility to be infected by fluke, which would not necessarily be the case if there was no fluke on the farm. R (R Core Team, 2011) was used, with the lme4 (Bates et al., 2015) and ggplot2 (Wickham, 2009) packages. Due to confidentiality agreements relating to FSS/FSA project FS101055 which funded the faecal sample collection, figures or data relating to groups of fewer than five farms cannot be shown.

### 2.5 Multilevel model

Correlations between the numerical explanatory variables were checked to ensure highly correlated variables were not entered simultaneously into the model. All models were fitted using maximum likelihood. Linear and logistic regression models were built with log_10_ *E. coli* cfu/g and a positive *E. coli* result respectively as the outcome variable. Either log fluke ELISA PP or a positive fluke result was used as the only level 1 explanatory variable, and all other animal and farm management information were level 2 variables. One and 2 respectively were added to the *E. coli* O157 count and fluke ELISA PP results before logging to deal with zero and negative values.

The starting point was a variable intercept model including a positive fluke result as a level 1 explanatory variable and farm as a level 2 random effect. Management variables which met the inclusion criteria were then added one at a time. A seasonal pattern was expected for *E. coli* (Ferens and Hovde, 2011), so day was modelled as a sinusoidal function to allow for this. The same process was repeated with fluke PP as the explanatory variable. The process was then repeated again with log_10_ *E. coli* cfu/g as a continuous outcome variable. Variable slopes were also tested. The Akaike information criterion (AIC) was used to compare models, with a lower AIC considered better than a higher one.

## 3 Results

Between 13th January and 19th October 2015, of 39 farms sampled with one or more cattle testing positive for *E. coli* O157, two declined to take part in further research and samples from two farms were delayed in transit and were therefore not suitable for fluke testing. There was insufficient sample for testing from a further five cows. Therefore, samples from 810 cattle from 35 herds were tested using the *F. hepatica* copro-antigen test. Of these, 14 farms had at least one cow testing positive for *F. hepatica.* Between 7 and 40 cattle were sampled from each of these farms (median = 22, total = 334) and are included in the following analysis.

### 3.1 Descriptive statistics

The characteristics of the farms are shown in Table 1. The data were examined to find out whether groups of cattle were housed or grazing, how long they had been housed or grazing for, and whether they had received a worming or flukicide treatment within the past 3 months. However, even in groups for which flukicide use was recorded, fluke copro-antigen ELISA positive cattle were still present, and similarly some groups of cattle which had been housed for several months still had significant numbers of fluke positive animals. Therefore treatment history was not used to exclude farms and all animals that came from groups with at least one fluke case were included, on the basis that they would all have had the chance to become fluke infected.

#### 3.1.1 Animal level

**Fig. 1:**
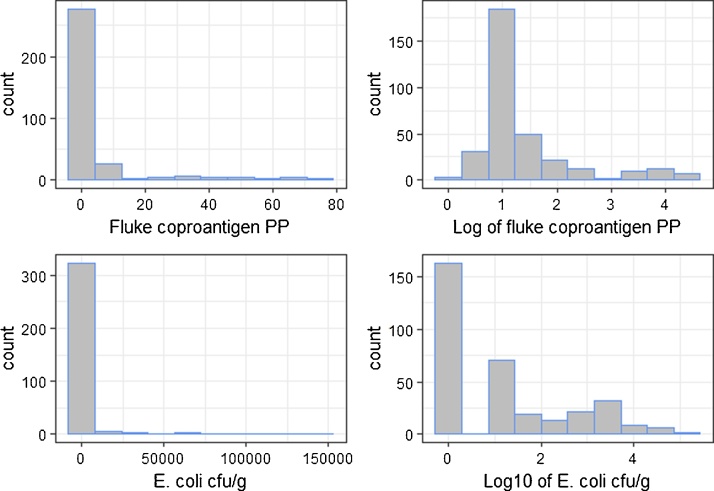
Distribution of *F. hepatica* coproantigen PP values and *E. coli* O157 cfu/g for animals across all farms.

Overall, 50.9% of cattle tested positive for *E. coli* O157 and 13.2% tested positive for *F. hepatica.* The distributions are shown in Fig. 1.

#### 3.1.2 Farm level

Within farms, between 4 and 100% of cattle tested positive for *E. coli* (mean = 43.5%, median = 43.1%) whilst for *F. hepatica* the range was 2.1–100% (mean = 14.7%, median = 6.5%). The distribution of log *E. coli* O157 cfu/g varied between farms, but in general it was right skewed in ten farms whilst four farms showed a more symmetrical platykurtic distribution. For fluke PP, all except one farm had a right skewed distribution.

The farms were spread throughout Great Britain with six from Scotland, four from England and four from Wales. North Wales, South Wales, the Welsh borders, Northern England and a variety of Scottish locations were represented.

### 3.2 Associations between fluke and *E. coli* O157

Inspection of scatterplots revealed no visible association between the fluke PP and log *E. coli* O157 cfu/g, either at individual or farm level (data not shown). More detailed inspection of three farms with more than 10% of cattle testing positive for fluke revealed no consistent pattern with regard to which individuals were positive for which pathogen. In one farm all of the fluke positive animals were also *E. coli* positive, in a second farm all of the fluke positives were *E. coli* negative, and in a third farm all animals had fluke and the PP values were evenly spread between the *E. coli* positive and negative animals.

### 3.3 Multi-level models

The plotting of management variables against log_10_ *E. coli* O157 cfu/g did not reveal any non- linear relationships. No correlations of r > 0.7 were seen between any of the explanatory variables, except between numbers of animals of different ages/types.

Four different combinations of output and input variables were tested, to include all combinations of the *F. hepatica* and *E. coli* data. The inclusion of random effects improved the model fit in every case, indicating that there were important differences between farms. The explanatory management variables shown in Table 1 were each added to the model as level 2 variables, but it was not possible to add more than two variables at once because of the relatively small number of fluke cases, which led to non-convergence of the model due to perfect partitioning.

The best models for each combination of *E. coli* O157 positive and log_10_ *E. coli* O157 cfu/g, and log fluke PP and fluke positive are shown (Table 2). The fluke result did not explain any additional variation in three out of four models, however, log fluke PP was significant when modelled against positive *E. coli* O157 result. Day of sampling and the age of the youngest animal in the group were included in all of the models at level 2 and were highly significant in all models (*p <* 0.0001). The higher the age of the youngest animal in the group, the lower the odds of infection with *E. coli* O157. The model fitted better with day of sampling as a linear variable, and the odds of *E. coli* O157 was found to decrease throughout the year, from January until October. The introduction of random slopes worsened the model fit in each case so this was not pursued.

**Table 2:** Summary of the multi-level models. Farm was included as a random intercept. Day of sampling and age of the youngest animal in the group were controlled for in all models.

Outcome variable	Input variable	Co-efficient	*p* value
*E. coli* O157 positive	*F. hepatica* positive	0.50	0.34
*E. coli* O157 positive	Log *F. hepatica* ELISA PP	0.48	0.010
log_10_*E. coli* O157	*F. hepatica* positive	−0.02	0.90
log_10_*E. coli* O157	Log *F. hepatica* ELISA PP	0.09	0.26

*E. coli* O157 positivity was determined using immune-magnetic separation. log_10_*E. coli* O157 refers log_10_ of the *E. coli* O157 count (cfu/g). *F. hepatica* positivity was determined using a copro-antigen ELISA. ELISA PP is the percentage positivity compared to a known positive sample.

## 4 Discussion

This study aimed to use samples available from the FSS/FSA study to investigate whether shedding of *E. coli* O157 is associated with *F. hepatica* infection in cattle (Hickey et al., 2015). *E. coli* O157 is the serogroup most commonly detected in humans in the UK, Europe and the US, and is associated severe clinical outcomes in humans (Anon, 2015, Browning et al., 2016). The advantage of using the samples from a pre-existing study was efficiency in terms of reducing resources needed for planning, recruitment of farmers, visiting farms and testing samples for *E. coli* O157. However, the biggest disadvantage of using the samples gathered for the FSS/FSA study was that the sampling method was designed to treat the group of cattle as the unit of interest; specifically, to identify groups where at least one animal was shedding *E. coli* O157 (Gunn et al., 2007, Pearce et al., 2009). *E. coli* O157 shedding varies widely from day to day (Robinson et al., 2009), and the effective sensitivity may be as low as 40% for a one-off faecal sample (Echeverry et al., 2005). Whilst this was not a problem for the FSS/FSA study, where the group was treated as the epidemiological unit, it may have affected the current study because individuals that were shedding *E. coli* O157 may have been missed.

Reaching the required sample size depended on the initial assumptions about prevalence of the two pathogens being reasonably accurate, particularly as the collection of additional samples was not possible given the constraints of the study. However, the levels of fluke infection seen in this study, both at the farm and the animal level, were much lower than had been assumed for the sample size calculations (Hickey et al., 2015), and *F. hepatica* only occurred in 43% of farms compared to the predicted 70–80% (Salimi-Bejestani et al., 2005a, McCann et al., 2010, Hickey et al., 2015). This lower fluke prevalence may be partly explained by differences between the cattle populations that were the subject of the FSS/FSA study and previous studies. Data on herd level prevalence of infection is from lactating dairy cows, whereas the FSS/FSA study sampled mostly beef breed or cross bred store or finishing animals. Differences in management exist between these groups that are likely to affect their risk of fluke infection. For example, treatment for fluke is more difficult in dairy animals due to the long milk withhold times of flukicides. Also, of the 35 groups of cattle tested for fluke, only nine were currently grazing, and of those, three had been turned out onto pasture within the three weeks prior to sample collection. This could have been due to the time of year when the samples were collected but also the nature of the farming units tested. It is possible that some of the groups were permanently housed, which would put them at low risk of fluke exposure, although this information was not available from the questionnaire.

The lower than expected prevalence of fluke could also be due to the relatively low sensitivity of the *F. hepatica* copro-antigen test, which in naturally infected cattle has been estimated to be below 50%–60% (Duscher et al., 2011), whereas the bulk milk tank antibody ELISA used to estimate prevalence in previous studies has a sensitivity of 96% (Salimi-Bejestani et al., 2005a). The difference in sensitivity between the two types of test was not taken into consideration in the feasibility study (Hickey et al., 2015). The relatively low sensitivity of the diagnostic tests used for both fluke and *E. coli* could have led to non-differential misclassification. This is expected to bias the observed effect size towards zero, although sometimes, by chance, the effect size can be over-estimated (Jurek et al., 2005).

Delays in implementation of this study led to the loss of samples from 17 farms enrolled in the FSS/FSA study that were sampled between September 2014 and January 2015 and which had agreed to take part. This contributed to the failure to reach the required sample size. The missed samples were taken during the season when the within-herd prevalence of fluke might have been expected to be at its highest (Bloemhoff et al., 2015).

In spite of these challenges, one of our models showed a significant association between *F. hepatica* and *E. coli* O157. This would be consistent with our initial hypothesis that *F. hepatica* mediated down-regulation of Th-1 immunity may limit the ability of cattle to clear *E. coli* O157 from the intestinal tract: indeed, this would be similar to the previous observation that *F. hepatica* infections in cattle result in increased susceptibility to *Salmonella dublin* which is associated with reduced cellular immune responses against the bacteria (Aitken et al., 1979).

The cut-off of the fluke copro antigen ELISA has been the subject of debate, with some studies setting their own cut-off to increase sensitivity (Brockwell et al., 2014). A continuous measure of PP avoids this problem and PP is a biologically meaningful measure as antigen level is correlated with fluke burden (Kamaludeen, 2016).

The addition of more than two additional explanatory variables was prevented by insufficient variability within the data. This could partly explain the observed large random effect of farm, which indicates that there were large differences between farms. Another interesting question is whether the inter farm differences could be partially explained by differences between strains of *E. coli* O157 at the molecular level that might be related to shedding events and immune status. Indeed it is known that different strains of *E. coil* O157 induce different types of immune response (Corbishley et al., 2014) and different genetic traits of *E. coli* O157, such as phage type (Chase-Topping et al., 2007), presence of *stx2a* and *stx2c* genes and polymorphisms in the *tir* gene (Arthur et al., 2013), are associated with either high or low shedding from infected individuals. Although it was important to control for day of sampling, as season is associated with observed prevalence of both fluke and *E. coli* O157 (Bloemhoff et al., 2015, Ferens and Hovde, 2011, Smith et al., 2016, Synge et al., 2003), the strong effect seen here is more likely to be due to all animals from a single farm being sampled on the same day than a genuine seasonal effect. Therefore caution should be used when interpreting the direction and size of the seasonal effect. There may also be other explanatory or confounding variables that are not included in these models. The result should therefore be interpreted with caution, particularly as the effect size is small and it is only seen in one of the model combinations.

The results of our study hint at an association between *E. coli* O157 shedding and *F. hepatica* infection that merits further investigation. Based on our experiences, use of pre-collected samples represents a cost–effective way of obtaining data, however, care needs to be taken to avoid certain pit falls. In the planning stage of future studies, worst case scenarios for prevalence should be considered, taking into account diagnostic test accuracy and differences between populations which may affect apparent prevalence. Even more importantly, efforts should be concentrated on ensuring that the true infection or shedding status of each individual can be ascertained, and that the type, number and size of sample are suitable for this. For *E. coli* shedding this is likely to include longitudinal sampling to address the issues of intermittent shedding and uneven distribution of bacteria within the faeces.

## Conflict of interest

None.
